# A Rare Metastasis to the Bladder

**DOI:** 10.1155/2013/789039

**Published:** 2013-03-03

**Authors:** Rishi A. Modh, Katherine A. Corbyons, Lawrence L. Yeung

**Affiliations:** Department of Urology, University of Florida, 1600 SW Archer Road, P.O. Box 100247, Gainesville, FL 32610, USA

## Abstract

Primary bladder cancer is the fifth most common malignancy but secondary malignancies of the bladder are rare. Distinguishing primary adenocarcinomas of the bladder from secondary adenocarcinomas is difficult and relies on immunohistochemical staining. Prostate, colorectal, breast, and lung all can produce metastatic adenocarcinomas to the bladder. Further management of the malignancy varies depending on the source, thus making proper diagnosis critical. We present only the fifth documented case of metastatic adenocarcinoma of the lung to bladder and performed a review of the literature.

## 1. Introduction

Bladder cancer is the fifth most commonly diagnosed malignancy. An estimated 73 510 individuals will be diagnosed with bladder cancer in 2012. Most bladder cancers are diagnosed at an early stage (non-muscle-invasive), but up to 25% present at more advanced, invasive stages [1]. The majority of primary cancers of the bladder are pure urothelial while squamous cell, small cell, and glandular or adenocarcinoma account for a small percentage. Urothelial carcinomas can have mixed histologic variants including squamous, glandular, sarcomatoid, micropapillary, small cell, and plasmacytoid. Rare histologic subtypes of primary bladder cancers include small cell, sarcomatoid, and pheochromocytoma [2]. 

Secondary cancers to the bladder are also rare. They are often categorized as direct extension of tumor from surrounding organs, metastasis, and lymphoma/leukemias. The most common sites of spread to the bladder are prostate, colorectal, or cervical sites. Breast, lung, and skin primaries are less common sources. Diagnosis and eventual treatment rely on the patient's history, the surgical specimen, and immunohistochemical staining. Primary adenocarcinomas of the bladder account for approximately 2% of bladder tumors and usually involve the embryological remnant of the urachus [3, 4]. Differentiating primary adenocarcinomas of the bladder and metastatic adenocarcinoma lesions can be difficult and relies heavily on pathologic evaluation. We present an unusual case of metastatic adenocarcinoma of the lung to the bladder.

## 2. Case Report

An 81-year-old female with a 50-pack-year history of smoking was initially referred to the neurosurgical clinic for left carpal tunnel syndrome. She underwent carpal tunnel release but her pain progressed to her left shoulder. A chest X-ray revealed a new atypical density in the left upper lobe measuring 5 × 2.5 cm. A followup CT and PET scan demonstrated two lung masses suspicious for malignancy and three small nodules in the right lung that were suspicious for metastatic disease. MRI examination revealed a Pancoast tumor extending from the left lung apex to the brachial plexus and into the vertebral bodies and neural foramina from C6 through T1. She eventually underwent a CT-guided biopsy that was immunoreactive for TTF-1 and CK7, suggesting adenocarcinoma of the lung as the primary source.

**Figure 1:**
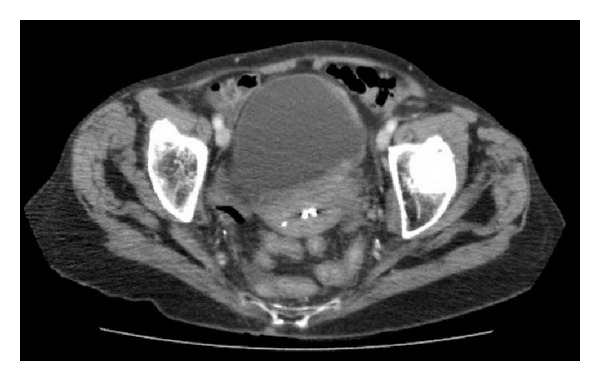
Abdominal and Pelvic CT showing a thickened posterior and left lateral wall.

**Figure 2:**
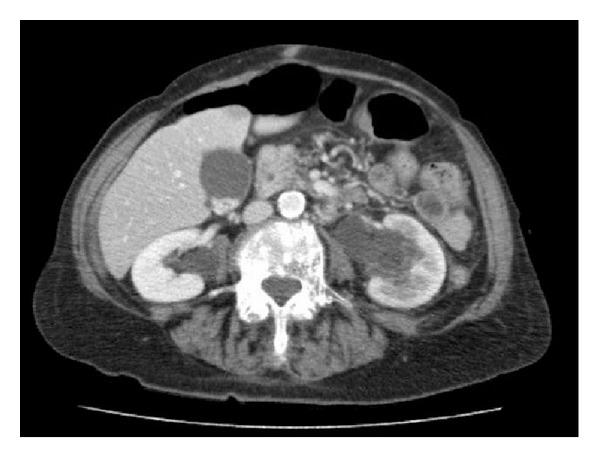
Abdominal and Pelvic CT showing bilateral hydronephrosis.

Six months after completion of chemotherapy, she developed three days of worsening abdominal pain and a deterioration in her renal function. A CT scan revealed bilateral hydronephrosis and thickening within the left lateral and posterior bladder (Figures 1 and 2). She eventually underwent cystoscopy, transurethral resection of the concerning area, and ureteral stent placement. The bladder mucosa appeared normal on exam with extrinsic compression into the lumen of the bladder. Urine cytology was negative for malignancy and she never developed gross hematuria. 

The final pathology revealed adenocarcinoma involving the muscularis propria with benign urothelial mucosa with reactive changes. Immunophenotypically, this was most compatible with a lung primary. The tumor was immunoreactive for CK7 and Napsin A, partially immunoreactive for p53, and nonreactive for CK20, TTF-1, Surfactant B, and estrogen receptor (ER).

## 3. Discussion

In order to differentiate metastatic adenocarcinoma of lung from primary adenocarcinoma of the bladder, immunohistochemical studies are required. The original diagnosis of adenocarcinoma of the lung was dependent on TTF-1 positivity. However, in our case, TTF-1 was negative while Napsin A and CK7 were positive. A previous case report was able to distinguish a patient's new primary adenocarcinoma of bladder with previously diagnosed primary adenocarcinoma of the lung. In this case, the patient's bladder specimen was negative for Napsin A and TTF-1 but was positive for more bladder-specific stains including GATA3 and S100P [5]. Napsin A has been shown to be a reliable marker to distinguish adenocarcinoma of the lung from primary adenocarcinoma of the bladder. It is more sensitive and specific for primary adenocarcinoma of the lung than TTF-1 [6]. These immunohistochemical stains, along with the pathologic specimen predominately involving the outer layers of the bladder muscle while sparing the mucosa, provide strong evidence of metastatic spread of a primary adenocarcinoma of the lung. 

To our knowledge, only four prior cases of primary adenocarcinoma of the lung with metastatic spread to the bladder have been identified in the literature. The first described case was a single patient in Spain, where the authors described an intact urothelium at endoscopic resection with the tumor affecting the lamina propria and containing the same immunologic characteristics of the patient's previous adenocarcinoma of the lung [7]. 

The next case was seen in a review of 282 secondary lesions of the bladder. In this review, secondary lesions represented on 2.3% of all bladder tumors and the majority were from colorectal or genitourinary primaries. Eight of these were identified as primary lung and only one was deemed as adenocarcinoma of lung [8]. 

In Japan, a case of adenocarcinoma of the lung with metastasis was made after the bladder biopsy specimen was positive for TTF-1, and CK7 and negative for CK20. Interestingly this case also noted normal appearing bladder mucosa and primary involvement of the bladder muscularis [9]. 

Finally, a group in France described a patient with adenocarcinoma of the lung who presented with hematuria. The biopsy specimen again was positive for CK-7 and TTF-1 without any expression of CK-20 [10]. 

While rare, metastatic adenocarcinoma of the lung is important to distinguish from primary adenocarcinomas of the bladder, especially when cystoscopic examination of the bladder does not correlate with diagnostic imaging findings. This diagnosis relies on pathologic evaluation and immunohistochemical staining. Distinguishing between the two entities is critical to proper management of the respective malignancy.
